# Strengthening policy coding methodologies to improve COVID-19 disease modeling and policy responses: a proposed coding framework and recommendations

**DOI:** 10.1186/s12874-020-01174-w

**Published:** 2020-12-08

**Authors:** Jeff Lane, Michelle M. Garrison, James Kelley, Priya Sarma, Aaron Katz

**Affiliations:** University of Washington School of Public Health, Seattle, WA USA

## Abstract

**Background:**

In recent months, multiple efforts have sought to characterize COVID-19 social distancing policy responses. These efforts have used various coding frameworks, but many have relied on coding methodologies that may not adequately describe the gradient in social distancing policies as states “re-open.”

**Methods:**

We developed a COVID-19 social distancing intensity framework that is sufficiently specific and sensitive to capture this gradient. Based on a review of policies from a 12 U.S. state sample, we developed a social distancing intensity framework consisting of 16 domains and intensity scales of 0–5 for each domain.

**Results:**

We found that the states with the highest average daily intensity from our sample were Pennsylvania, Washington, Colorado, California, and New Jersey, with Georgia, Florida, Massachusetts, and Texas having the lowest. While some domains (such as restaurants and movie theaters) showed bimodal policy intensity distributions compatible with binary (yes/no) coding, others (such as childcare and religious gatherings) showed broader variability that would be missed without more granular coding.

**Conclusion:**

This detailed intensity framework reveals the granularity and nuance between social distancing policy responses. Developing standardized approaches for constructing policy taxonomies and coding processes may facilitate more rigorous policy analysis and improve disease modeling efforts.

**Supplementary Information:**

The online version contains supplementary material available at 10.1186/s12874-020-01174-w.

## Background

The first confirmed case of COVID-19 occurred in the United States (U.S) in Washington State on January 20, 2020 [1]. Non-pharmaceutical interventions, such as quarantines and mass social distancing, were the primary public health strategy for blunting COVID-19 spread. As confirmed case counts climbed, state, county, and municipal governments adopted policies recommending or requiring actions to reduce social density and slow the progression of the outbreak. The timing and intensity of social distancing policy responses has varied. Multiple efforts sought to rapidly code these social distancing policy responses for analysis [2–11]. Social distancing policy coding has been critical to COVID-19 disease models that have influenced policy decision-making whether to impose or ease social distancing approaches. For example, Dr. Deborah Birx, the U.S. Coronavirus Response Coordinator, has repeatedly cited the COVID-19 projections prepared by the University of Washington’s Institute for Health Metrics and Evaluation (IHME) [12]. However, the methods used by various modeling efforts for linking COVID policies to projected outcomes (e.g., rates of infection, hospitalization, or death), have been quite divergent.

Social distancing coding efforts have used a range of methodologies and frameworks to characterize and code policy responses, resulting in a diversity of social distancing policy taxonomies and classification schemes. These efforts have characterized social distancing into taxonomies consisting of 1 domain (e.g., stay at home order in place) to upwards of six domains. For example, McGrail et al. used a single domain of “lockdown,” coded as the date the lockdown started and ending as of the date non-essential retail stores re-opened [13]. In contrast, Adolph et al., which developed one of the first publicly available datasets, developed a COVID-19 framework originally consisting of five domains: (1) recommendations or restrictions on gatherings, regardless of the size of gathering; (2) K-12 school closures; (3) restaurant restrictions on in-person dining; (4) non-essential business closures; and (5) mandatory stay-at-home orders [2].

There is also a diversity in whether coding efforts have used binary or ordinal scoring within domains. Many methodologies have used binary coding to state whether a social distancing policy is in effect for a particular domain [2, 3, 5–10]. In contrast, some analyses have used ordinal scales within domains to illustrate gradients of social distancing within a domain (e.g., different levels of restaurant restrictions). For example, the dataset developed by Adolph et al. was subsequently updated to include intra-domain ordinal scales [4]. Hale et al. coded social distancing domains using non-standardized ordinal scales of 0–2, 0–3, or 0–4, depending on the domain [11]. Hale et al. also used a binary flag to adjust ordinal scale calculations when a policy has a targeted subnational geographic scope. Table 1 lists some of the social distancing policy coding efforts published to date and illustrates the diversity in policy coding methodologies used.

**Table 1:** Existing COVID-19 Policy Responses Coding Frameworks

Authors	No. of Domains	List of Domains	Intra-Domain Coding (binary or ordinal scale)	Data Source(s)
Adolph et al. [2]	5	gathering restrictions; school closures; restaurants; non-essential businesses; stay-at-home orders	Binary (later supplemented to include additional domains and some intra-domain scales)	Primary source policies
Krishnamachari et al. [3]	5	gathering restrictions; school closures; restaurants; non-essential businesses; stay-at-home orders	Binary	Adolph et al./Fullman et al. database
McGrail et al. [13]	1	lockdowns	Binary	Aura Vision Global COVID-19 Lockdown Tracker (which primarily relies on media reports)
Killeen et al. [5]	6	stay at home order; gathering ban greater than 50; gathering ban greater than 500; public school; restaurant dine-in; entertainment/gym	Binary	Media reports
Courtemanche et al. [6]	4	large event bans; entertainment-related businesses; school closures; shelter in place orders	Binary	Killeen et al. dataset
Fowler et al. [7]	1	Stay at home orders	Binary	New York Times website and local media reports
Abouk & Heydari [8]	6	statewide stay-at-home orders; stay-at-home orders only applying to certain populations or only certain counties or cities; non-essential business closures; large gathering bans; school closure mandates; restaurant and bar limits	Binary	Primary source policies collected by Kaiser Family Foundation “State Data and Policy Actions”
Strickland et al. [9]	3	stay at home orders; non-essential services closed; educational facilities closed	Binary	UW IHME website
Lasry et al. [10]	5 (statewide policy domains) 6 (local policy domains)	limits on mass gatherings; limits on senior living facilities; school closures; limits on bars and restaurants; stay-at-home/shelter-in-place orders; curfews	Binary	Primary source policies and media reports
Hale et al. [11]	6	school closing; workplace closing; cancel public events; restrictions on gatherings; close public transport; and stay at home requirements	Ordinal scale (0–2; 0–3; or 0–4)	Media reports; primary source policies

As of April 27, 2020, U.S. states began lifting social distancing requirements. The “re-opening” process often occurred in predefined stages leading to a gradient in social distancing that is hard to capture with a limited number of policy domains or a lack of a scale within domains. To help address such a fluid and complex policy environment, we sought to develop a COVID-19 policy coding framework that is sufficiently specific and sensitive to capture the gradient in social distancing policy responses during phases of “closing” and “re-opening.” We also sought to identify methodological recommendations to strengthen the COVID-19 comparative policy analyses to better inform research, disease modeling, and policy decision-making.

## Methods

We conducted a comparative content analysis of social distancing policies adopted by a sample of U.S. states. We purposively selected the first twelve U.S. states to reach 100 confirmed COVID-19 cases (New York, Washington, California, New Jersey, Massachusetts, Louisiana, Florida, Colorado, Illinois, Georgia, Pennsylvania, Texas). All of these states reached 100 confirmed cases by March 17, 2020. The total population of these 12 states represented almost 55% of the entire population of the United States [14].

Policies were identified by a search of public websites operated by state governments (e.g., governor or state department of health), and in some cases facilitated by reviewing the Kaiser Family Foundation list of state policy responses [15]. The inclusion criteria for policies included in the analysis were: (1) directive issued by the Governor or state agency lead regarding COVID-19 documented in an Executive Order or state government website, including but not limited to Department of Health; (2) the primary purpose of the policy is to reduce social density with the goal of reducing community transmission of COVID-19; and (3) policies issued between March 11, 2020 and June 19, 2020. June 19 was selected as the end date, because that date marked 100 days after the World Health Organization declared COVID-19 a pandemic and approximately 6 months after the first confirmed COVID-19 case in the United States, which occurred on January 20, 2020. We excluded from the analysis policies that were: (1) recommendations or directives issued orally only (e.g., at a press conference and not released as a press release on the governor’s website); (2) recommendations or directives issued by local governments (e.g., municipal or county government officials) or the U.S. federal government.

The content of responsive policies were analyzed using a multi-step process. First, a subset of state policies were reviewed to familiarize the reviewers with the content. Second, the highest level of the taxonomy framework was developed to capture differences in policy responses that may materially affect public health outcomes and to optimize sensitivity of the framework. The objective was to capture differences in the timing and intensity of social distancing policy responses through phases of “closing” and “re-opening.” We identified 16-domains where our sample indicated differences in policy approaches: social gatherings; religious gatherings; funerals; stay at home orders; restaurants; bars; movie theatres; hair salons and barbers; indoor gyms; non-essential retail stores; childcare; K-12 schools; higher education; nursing homes; prisons; and voting.

Third, an intra-domain scale was developed of 0–5 to align with the following framework: No Recommendations or Mandates; Recommendations Only; Mandates-Low; Mandates-Medium; Mandates-High; Mandates-Very High. Fourth, to improve inter-rater reliability, unique sub-domain scales were developed for each of the 16-domains to capture differences in policy approaches within each domain (e.g., different types of restaurant restrictions such as prohibiting on-premises dining but allowing takeaway orders versus allowing on-premises dining but with an occupancy cap).). This resulted in a taxonomy comprising 16 first level domains and 6 levels within each domain. Each of the sub-domains was assigned an ordinal value of 0–5. Under this scoring rubric, the range for daily intensity is 0–80. Results data are presented using average daily intensity (range: 0–5). The intensity scale is ordinal, meaning that the numeric relationship between levels of intensity is not necessarily proportional to the effect on social distancing. For example, an intensity scale of 4 in a particular domain does not necessarily mean that the magnitude of social distancing imposed by that level is twice as intense as a score of 2 in the same domain.

Policies were reviewed independently by two co-authors and extracted into an Excel sheet. The data were coded longitudinally in Excel by effective date of the policy (not the announcement date), by domain, and sub-domain (i.e., 0–5 scale within each domain). This allowed analysis and comparison of domain-specific policy responses or aggregated across all or subset of domains. The datasheets used for data extraction and coding are available for public review [16]. One co-author (JL) coded all 12 states with other co-authors coding a subset of states (JK, PS, and AK). Differences in coding were discussed and resolved via consensus among the coders. Interpretation notes were added to the Excel datasheet to document key interpretations. The domain-specific scale definitions were revised at points during the coding process to capture observed differences in policy approaches while maintaining the 0–5 scale for each domain. The data extraction sheet was reviewed throughout to ensure that coding was updated as necessary when a domain scale was revised.

The full social distancing intensity framework, including the detail of domain-specific scales, is included as Appendix A. We subsequently reviewed two public COVID-19 policy tracking databases developed by Kaiser Family Foundation [15] and Raifman et al. [17] to validate our findings and identify any potential discrepancies.

We identified a number of policies adopted by state-level government (e.g., governor Executive Order or Department of Health order) that only applied in a particular county or region within the state. These policies met our inclusion criteria and were included in our analysis. For the days a policy was in effect, we calculated a weighted daily average intensity within each domain by 1) coding the number of sub-regions at each intensity level on that day, 2) multiplied the intensity score for those sub-regions by the proportion of sub-regions with that intensity score, and 3) then summed the result.

$$ weighted\ daily\ average\ intensity\ within\ each\ domain=\sum \limits_{i=0}^5\frac{n_i}{n}\ast i $$

In this equation, i = intensity score, n_i_ = number of sub-regions with that intensity score, and n = total counties in the state. Our analysis and results treats all sub-regions equally. An alternative approach (not taken here) would be to multiply by the proportion of the state’s population living in counties with that intensity score in the second step instead of by the proportion of counties with that score. We elected not to adjust by population in part due to uncertainty of population levels affected by different sub-regions. These policies impact not only where people live (which can be ascertained via census data) but also where people work, shop, and recreate – and the latter categories are far more complex to validly ascertain, especially across states. The overall daily average intensity score is then the mean of the 16 domain-specific scores.

Following completion of coding, we conducted descriptive analyses of longitudinal change in social distancing policy intensity. We collected longitudinal, confirmed case count and mortality data from the Johns Hopkins University COVID Tracker database for data visualization purposes only. As the impact of policy on outbreak progression is not immediate, our data visualizations depict COVID-19 outcomes in relation to the concurrent policy intensity (i.e., the policy status on the same day as the outcomes) and the policy intensity as of 2 weeks prior to incidence rate outcomes to visualize an estimated lag between policy adoption and potential impact. Infectious outcomes such as incidence and mortality rates can be presented as daily, weekly, monthly, or yearly rates. While daily rates can experience a lot of variability in day-to-day fluctuations – some of which may be driven in part by scheduling or other procedural issues – monthly rates may not be “real time” enough to pick up the impact of policy changes. To find a middle ground, here we used rolling 7-day averages – so the rate for each day is the incidence for the 7 days of which that date is the midpoint. While we would typically constrain all vertical axes to the same maxima if the goal was to directly compare incidence and mortality rates across states, here the goal is to illustrate the relationship between policy intensity and subsequent incidence. As a result, we constrained the right-hand policy vertical axis to the maximum daily average policy intensity score but left the left-hand incidence value vertical axis unconstrained.

## Results

Table 2 provides a summary of key data points from the results of the analysis. As Table 2 illustrates, the states from our sample with the highest average daily intensity were Pennsylvania (4.19), Washington (4.13), Colorado (4.13), California (4.06), and New Jersey (4.06). The states from our sample with the lowest peak average daily intensity were: Georgia (2.94), Florida (3.25), Massachusetts (3.56), and Texas (3.63). The states with the highest daily average intensity on June 19 were: New York (3.47), New Jersey (3.44), Washington (3.15), and California (2.95). The states with the lowest daily intensity on June 19 were: Florida (2.08), Georgia (2.19), Texas (2.50), and Louisiana (2.56).

**Table 2:** Social Distancing Intensity by State

State	Date of First Mandate^a^	Peak Daily Intensity^b^	Date Range of Peak Intensity^c^	No. of Days Between First Mandate and Peak Intensity	No. of Days at Peak Intensity	Intensity on June 19
California	3/11	4.06	3/24–5/7	13	44	2.95
Colorado	3/12	4.13	3/27–4/26	15	30	2.88
Florida	3/14	3.25	4/3–5/3	20	30	2.08
Georgia	3/18	2.94	4/3–4/22	16	19	2.19
Illinois	3/11	3.75	3/26–4/30	15	35	2.75
Louisiana	3/12	3.69	4/9–5/14	28	35	2.56
Massachusetts	3/13	3.56	3/24–5/17	11	54	2.94
New Jersey	3/13	4.06	4/1–5/12	19	41	3.44
New York	3/11	3.94	3/27–5/14	16	48	3.47
Pennsylvania	3/16	4.19	4/10–5/7	25	27	2.72
Texas	3/13	3.63	4/13–4/30	31	17	2.50
Washington	3/10	4.13	4/15–5/4	36	19	3.15

^a^The date on which at least one domain in the state was coded 2 or higher (i.e., Mandate-Low)

^b^The highest daily average intensity reached by the state during the sample period

^c^The date that the state reached its peak intensity and the last date the state was at its peak intensity before average intensity began decreasing

**Fig. 1:**
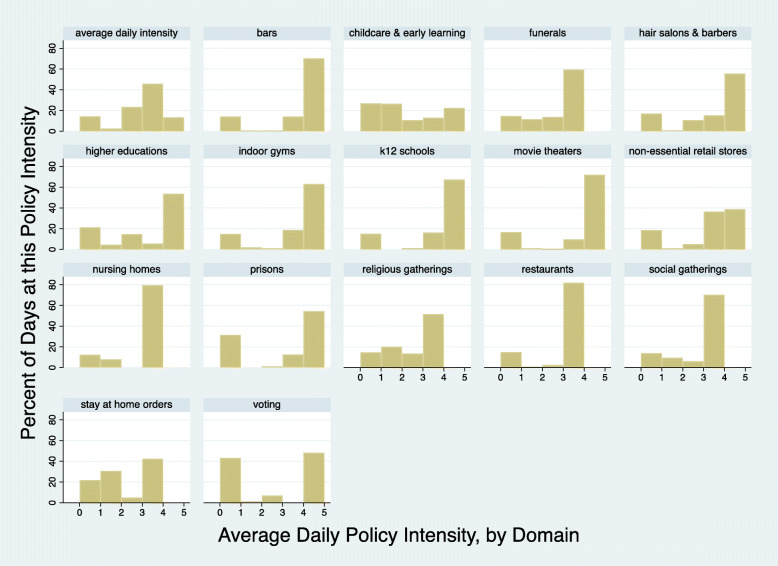
Distribution of Policy Intensity Scores, by Domain

Figure 1 shows the distribution of days at different policy intensity scores within each domain, looking at all states together. While not every possible sub-domain score was coded in our 12-state sample (e.g., no states in our sample reached a level 5 for stay-at-home orders), we maintained the 0–5 scale for all domains, in part, to allow for a Very High level (i.e., score of 5) in each domain. Maintaining the 0–5 scale gives the scale more flexibility for use in other locations where more stringent measures have been imposed (e.g., Lombardy, Italy) and allows for the framework to be used in the event more stringent measures are imposed in the U.S. in the future. For domains showing sharply bimodal distributions (such as restaurants or movie theaters), the added granularity of this policy coding structure may not add as much informational depth as it does for domains with broader distributions (such as childcare and religious gatherings). However, as states began to “open back up” we saw increased policy specificity even within domains such as restaurants and movie theaters, suggesting that we might expect to see broader distributions across all domains in the future.

**Fig. 2:**
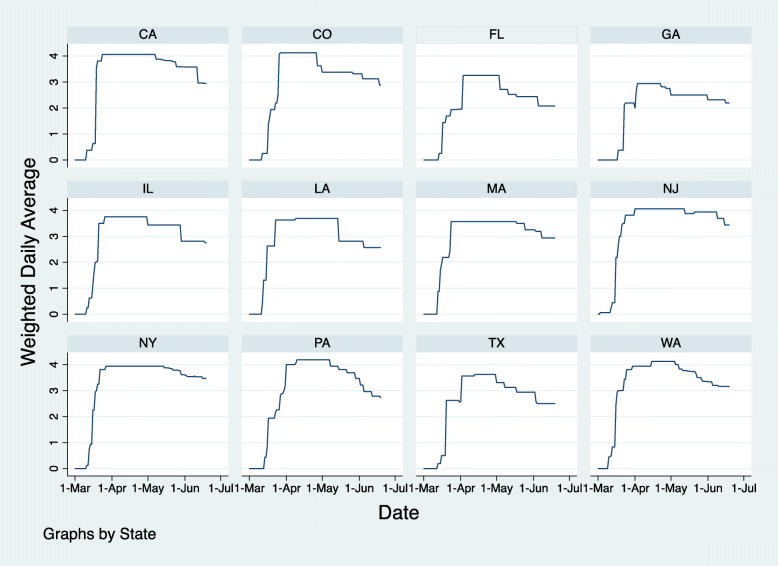
Average Policy Intensity Score, by Date and State

Figure 2 shows the results of the coding displayed for each state in our sample. As illustrated by Fig. 2, the slope of intensity change during the implementation of social distancing is more severe than during the easing of social distancing restrictions during “re-opening” up through June 19, which is more gradual and reflects the staged re-opening process used by many states.

**Fig. 3:**
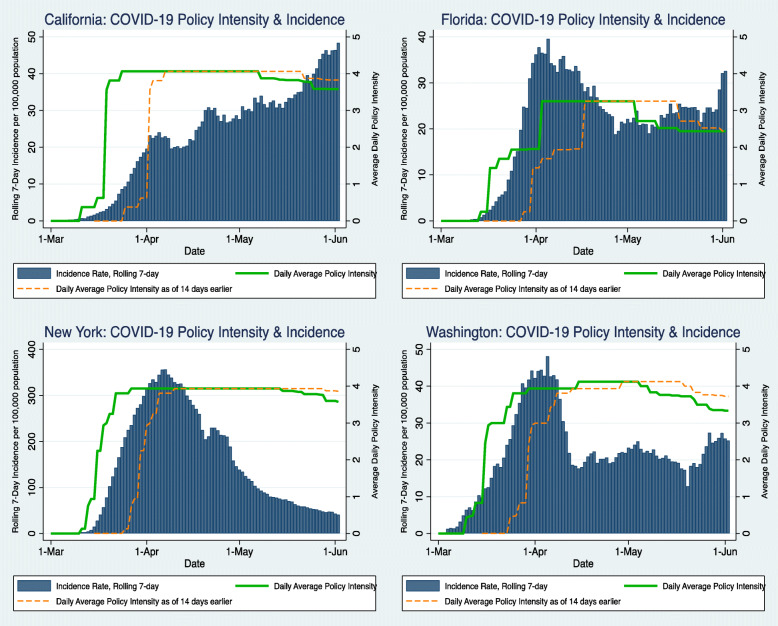
Average Daily Policy Intensity and COVID-19 Incidence Rates: A Four State Comparison

As can be seen in Fig. 3, our descriptive analysis reveals an apparent direct temporal relationship between increased policy intensity and a decrease in incidence rates in some states (such as Florida, New York, Washington) but not in others (such as California) – and the potential for a subsequent rise in incidence rates as policy intensity begins to drop back down (Washington, Florida). Even in those states which do show drops in COVID-19 incidence rates consonant with the intended policy effects, the relationship appears non-linear, with no decrease in transmission until a policy threshold is met. These graphs also help demonstrate why visualizing the potential lag time in policy impact can be helpful – with the green lines showing the average policy intensity in the state as of the same day as the incidence data, and the orange dashed line showing what the average policy intensity had been 14 days earlier when it had the potential to have an impact on the COVID-19 incidence rate of the current date. As not all policies will have equivalent lag times, it may be useful to visualize a range of different lag times to best identify patterns and relationships.

## Discussion

We sought to develop a COVID-19 policy coding framework that is sufficiently specific and sensitive to capture the gradient in social distancing policy responses during phases of “closing” and “re-opening” and to identify recommendations for strengthening policy coding methods for COVID-19 and other diseases. The 16-domain social distancing intensity framework presented here appears to capture the nuances of social distancing policy responses to COVID-19. This framework builds on the work of a number of social distancing frameworks [2–11]. The framework presented here may be especially well-suited to capture stages of re-opening and/or closing, which may call for iterative changes within domains. This framework may also be well-suited when comparing jurisdictions using different stage frameworks or when trying to isolate the relative contributions of different aspects of social distancing policies when a granular picture of differences in approaches may be needed.

A number of analyses have begun to assess social mobility data and their correlation with COVID-19 incidence. The detailed picture of policy status provided by this 16-domain social density intensity framework may also be critical for understanding fluctuations in social mobility resulting from iterative changes to social distancing policies during phased re-opening or closing.

An effective policy taxonomy should be sufficiently specific and sensitive to facilitate cross-jurisdiction comparisons of policy approaches, monitor material changes to policy frameworks over time, and improve our ability to identify policy variables that improve the relative performance of differing approaches [18]. Taxonomies dig beneath the titles of policies and highlight the underlying and discrete mechanisms of action. The phased re-opening plans of states within our sample illustrate the need for rigorous policy analysis and coding to inform policy decision-making. For example, New York is using a re-opening framework of 4 “Phases,” Pennsylvania is using a Red, Yellow, Green reopening framework, and California is using a series of “Stages.” The relative intensity of each stage/phase/color can vary between states. For example, indoor dining can restart in Washington State in counties reaching Phase 2, but indoor dining did not start in New York regions until they were designated as Stage 3.

The results of the data visualization illustrate the potential advantage of using a policy coding framework that captures nuanced differences in policy approaches. We are also beginning to see similar policy differences in masking policies. Evidence suggests that individual masking may substantially reduce COVID-19 transmission, and states are adopting a range of different types of policies with the goal of increasing individual masking in the population [19]. Some of these policies only recommend wearing masks in public, while other mandate that certain employees wear masks, and still others mandate that members of the public wear masks. Masking policy was outside the scope of our analysis, but the results of our analysis indicate that masking policies, like social distancing policies, should be analyzed in a way that captures the differences in these policy approaches. For example, a tiered masking policy coding framework has been developed by Fullman et al. [4]

A nuanced policy taxonomy will help us more accurately compare the differences between state policy approaches and potentially allow us to isolate approaches that have the most substantial effect, while minimizing social and economic harm. The coding taxonomy presented here provides one approach to capture the nuances of policy responses that may be important for monitoring differences between states as they respond to future outbreak fluctuations.

The policies we analyzed are limited to those adopted by state-level executive authorities (e.g., governors or departments of health); many local jurisdictions have adopted their own measures to institute social distancing sooner or more intensely than required by state authorities. We recorded an increase in the use of county-specific social distancing in a number of our sample states (i.e., Washington, Florida, New York, California, Pennsylvania), especially when they began easing social distancing requirements. For this reason, it may be important to monitor social distancing policy responses at the county-level to have the most accurate view of the current status of social distancing approaches in the United States. Doing so, however, must recognize the interplay between state and local policies and powers (e.g., some state executive orders prohibit more stringent local orders, while other allow them).

We included in our coding framework domains that have not been included in some other social distancing approaches, including domains for voting and prisons. These domains were harder to code using an ordinal scale. Voting presented challenges, because only some states had statewide elections scheduled for March, April, or May. In addition, some states, such as Washington State, already used all-mail in voting prior and unrelated to COVID-19, while others instituted or expanded mail-in balloting as a way to promote social distancing in direct response to the COVID epidemic. Prisons presented other challenges, because it was more difficult to order the relative intensity of some actions (e.g., prohibiting visitors vs. suspending intake from country jails). Nevertheless, the authors believe these are important domains to analyze for comparative analysis purposes, and we have included them in this proposed scale. An alternative approach for this domain could be to use a normal scale for some of these domains (e.g., 1 point if a sub-domain action is present), which would not require assigning a relative order of intensity, but would still provide a more nuanced picture of the response within that domain.

Another result of having a larger number of domains is that some domains were subject to social distancing together or indirectly by some state policies, while other states had more specific policy guidance that specifically identified different types of locations. For example, in some states, social distancing at commercial businesses was restricted through a ban on gatherings of certain size or stay at home order (e.g., California), while others ordered specific types of businesses to close in addition to issuing a stay at home order (e.g., Louisiana).

A limitation of this type of policy content analysis is that we did not assess the extent of implementation or enforcement of these policies. Implementation of public policies often varies from the written word for a variety of reasons, including limited enforcement resources or local interpretation. This may be especially true for some social distancing policies, such as bans on religious services, which rely on broad public compliance with very little enforcement by government officials. For this reason, cultural views of the importance of social distancing are important mediators for implementing the social distancing policies we analyzed. The effectiveness of social distancing policies may also change over time as people accommodate or, alternatively, grow tired of the restrictions. Other COVID-19 analyses are seeking to look at the connection between social mobility and incidence and overlaying social mobility data with granular policy data may help provide a more detailed picture of the interplay between policy adoption, compliance, and public health outcomes [20, 21]. Additional analysis of the effect of social distancing policy on actual social behavior will be critically important as policy makers decide whether and how to modify social distancing policy approaches in the future for COVID-19 and potentially for other infectious diseases.

Another limitation is that we have not yet assessed the predictive value of this coding framework compared to other frameworks, and this framework has not yet been used to populate disease modeling projections. The increased descriptive power of this framework may or may not yield greater accuracy or precision in disease models. Another limitation is that policy pronouncements were changing rapidly during this time period and state governments used different approaches for publicizing policy decisions. Therefore, it is possible that some policy decisions were not captured in this analysis. Reviewers also needed to interpret some policy documents regarding their applicability to certain domains. These interpretations are documented in our dataset, which is publicly available [16]. To mitigate inter-rater reliability issues and ambiguity in policy text, we had two reviewers review each set of policies and validated our findings and interpretations using two other public COVID-19 policy tracking databases [15, 17].

The rapid pace of COVID-19 social distancing analyses reveals the importance of developing standardized approaches for policy taxonomies and coding processes. Improved granularity and transparency will facilitate more rigorous analysis and improve disease modeling efforts that rely on sensitive prognostic variables, such as assumptions about future government policy responses. Based on our experience developing this coding framework, we make a number of recommendations for policy coding methods to improve comparative policy analyses and disease modeling for COVID-19 and other disease. First, as part of any policy coding process a taxonomy should be developed that captures the nuances and gradients between policy approaches (domains, sub-domains) that may have a material effect on the effectiveness of such policies. Researchers should consider an intensity or stringency ordinal scale when appropriate to facilitate comparison between jurisdictions but recognize that not all domains are necessarily appropriate for an ordinal scale. If an ordinal scale is not appropriate, consider a normal scale, which does not have an ordering. Policy coding efforts should clearly define scales and domains to improve inter-rater reliability and to facilitate updates as policy approaches evolve. Researchers should capture PDF and/or screenshots of policies when conducting reviews to ensure access even after websites have been updated or revised (especially for rapidly evolving policy responses, such as the responses to COVID-19).

Transparency and public review of policy coding efforts will be important for strengthening confidence and reliability of disease models. Researchers should identify specific policies relied upon (e.g., title, date) and, if feasible, make copies of those policies publicly available to allow secondary review and broader access to the primary source policies. To improve accuracy and equity in policy coding research, researchers should consider inviting co-investigators residing in the jurisdictions where the policy is in effect to help validate policy interpretations (especially when multiple international jurisdictions are included). Researchers can encourage public review of coding and interpretation to help validate policy interpretations, and coding datasets should include interpretation notes so reviewers and other researchers are aware of how ambiguities were resolved or interpreted. A number of the COVID-19 policy coding efforts have set this example by making their underlying policy coding data publicly available [2, 4, 11], which we have also done [16].

## Conclusion

COVID-19 has presented one of the most rapid and intense phases of public health policy development and implementation in the last century. Often these policy decisions have had to be made with imperfect evidence under very tight timelines. As we continue to live with COVID-19, attempt to ease social distancing, and allow greater economic activity and social interaction, accurate disease models and policy monitoring systems will be critical to evidence-based policymaking. The effectiveness of state-level policies calling for social distancing may be influenced by a variety of factors, including public leadership, local/county policy responses, and local culture. Nevertheless, state-level policy decision-making is an important part of the story of social distancing in response to COVID-19. We hope this detailed social distancing intensity framework and the associated recommendations will provide a more granular view of social distancing approaches and contribute to improving the governmental response to COVID-19. We also hope this framework and recommendations will lead to additional collaboration across COVID-19 policy tracking and analysis groups and be supplemented over time to include other key components of the COVID-19 policy response (e.g., mandated masking). These methods and recommendations may also be useful for efforts to code other types of health policies to inform comparative analyses and modeling for a range of diseases and conditions.

## Supplementary Information

**Additional file 1.** Appendix A: Social Distancing Intensity Framework.

## Data Availability

The data referred to in the manuscript is available at the following website: https://openicpsr.org/openicpsr/project/120598/version/V1/view

## Abbreviation

COVID-19: Coronavirus disease 2019

## References

[1] Harcourt J, Tamin A, Lu X, Kamili S, Sakthivel SK, Murray J (2020). Severe Acute Respiratory Syndrome Coronavirus 2 from Patient with Coronavirus Disease, United States. Emerg Infect Dis.

[2] Adolph C, Amano K, Bang-Jensen B, Fullman N, Wilkerson J. Pandemic Politics: Timing State-Level Social Distancing Responses to COVID-19. medRxiv. 2020:2020.03.30.20046326. 10.1101/2020.03.30.20046326. 10.1215/03616878-8802162 32955556

[3] Krishnamachari B, Dsida A, Zastrow D, Harper B, Morris A, Santella A. Effects of Government Mandated Social Distancing Measures on Cumulative Incidence of COVID-19 in the United States and its Most Populated Cities. medRxiv. 2020:2020.05.22.20110460. 10.1101/2020.05.22.20110460.

[4] Fullman N, Bang-Jansen B, Reinke G, Magistro B, Amano K, Adolph C (2020). State-level social distancing policies in response to COVID-19 in the US Version 1.58.

[5] Killeen B, Wu J, Shah K, Zapaishchykova A, Nikutta P, Tamhane A, et al. A county-level dataset for informing the United States' response to COVID-19 Baltimore, MD: Johns Hopkins University; Available from: https://arxiv.org/pdf/2004.00756v1.pdf. Accessed 5 Aug 2020.

[6] Courtemanche C, Garuccio J, Le A, Pinkston J, Yelowitz A (2020). Strong social distancing measures in the United States reduced the COVID-19 growth rate. Health Aff (Millwood).

[7] Fowler JH, Hill SJ, Obradovich N, Levin R. The Effect of Stay-at-Home Orders on COVID-19 Cases and Fatalities in the United States. medRxiv. 2020:2020.04.13.20063628. 10.1101/2020.04.13.20063628.

[8] Abouk R, Heydari B. The Immediate Effect of COVID-19 Policies on Social Distancing Behavior in the United States. medRxiv. 2020:2020.04.07.20057356. 10.1101/2020.04.07.20057356. 10.1177/0033354920976575 PMC8093844 33400622

[9] Strickland CJ, Karaye IM, Horney JA. Associations Between State Public Health Agency Structure and Pace and Extent of Implementation of Social Distancing Control Measures. J Public Health Manag Pract. 2020. 10.1097/PHH.0000000000001215. 10.1097/PHH.0000000000001215 32487927

[10] Lasry A, Kidder D, Hast M, Poovey J, Sunshine G, Winglee K (2020). Timing of community mitigation and changes in reported COVID-19 and community mobility - four U.S. metropolitan areas, February 26-April 1, 2020. MMWR Morb Mortal Wkly Rep.

[11] Hale T, Noam A, Kira B, Petherick A, Phillips T, Webster S. Working Paper - Variation in Government Responses to COVID-19, Version 6.0: University of Oxford, Blavatnik School of Government; Available from: www.bsg.ox.ac.uk/covidtracker. Accessed 25 May 2020.

[12] Remarks by President Trump, Vice President Pence, and Members of the Coronavirus Task Force in Press Briefing, April 10, 2020 [Available from: https://www.whitehouse.gov/briefings-statements/remarks-president-trump-vice-president-pence-members-coronavirus-task-force-press-briefing-24/. Accessed 5 Aug 2020.

[13] McGrail DJ, Dai J, McAndrews KM, Kalluri R. Enacting national social distancing policies corresponds with dramatic reduction in COVID19 infection rates. medRxiv. 2020:2020.04.23.20077271. 10.1101/2020.04.23.20077271. 10.1371/journal.pone.0236619 PMC7392246 32730356

[14] United States Census Bureau Quick Facts: United States Census Bureau; Available from: https://www.census.gov/quickfacts. Accessed 3 Aug 2020.

[15] Kaiser Family Foundation State Data and Policy Actions to Address Coronavirus. Available from: https://www.kff.org/health-costs/issue-brief/state-data-and-policy-actions-to-address-coronavirus/. Accessed 5 Aug 2020.

[16] Lane J, Garrison M, Kelley J, Sarma P, Katz A (2020). Social Distancing Policy Intensity in U.S. States.

[17] Raifman J, Nocka K, Jones D, Bor J, Lipson S, Jay J, et al. COVID-19 US state policy database 2020. Available from: www.tinyurl.com/statepolicies. Accessed 5 Aug 2020.

[18] Organisation for Economic Development and Co-Operation. Proposal for a Taxonomy of Health Insurance, OECD Study on Private Health Insurance. Organisation for Economic Cooperation and Development; 2004.

[19] Esposito S, Principi N, Leung CC, Migliori GB. Universal use of face masks for success against COVID-19: evidence and implications for prevention policies. Eur Respir J. 2020;55(6):2001260. 10.1183/13993003.01260-2020. 10.1183/13993003.01260-2020 PMC7191114 32350103

[20] Benzell SG, Collis A, Nicolaides C (2020). Rationing social contact during the COVID-19 pandemic: transmission risk and social benefits of US locations. Proc Natl Acad Sci U S A.

[21] Badr HS, Du H, Marshall M, Dong E, Squire MM, Gardner LM (2020). Association between mobility patterns and COVID-19 transmission in the USA: a mathematical modelling study. Lancet Infect Dis.

