# Supplementary material for: Strengthening policy coding methodologies to improve COVID-19 disease modeling and policy responses: a proposed coding framework and recommendations
Source: BMC Med Res Methodol. 2020 Dec 8;20:298. doi: 10.1186/s12874-020-01174-w (PMC7721792; doi:10.1186/s12874-020-01174-w)
Supplement: Supplementary file 1 — Additional file 1. Appendix A: Social Distancing Intensity Framework. [file 12874_2020_1174_MOESM1_ESM.docx]

**Appendix A: Social Distancing Intensity Framework**

| **Domain** | **Level** | **Score** |
| --- | --- | --- |
| **Social Gatherings** | No recommendations or rules implemented for this domain | 0 |
| **Social Gatherings** | Recommendations Only | 1 |
| **Social Gatherings** | Mandates - Low (e.g., prohibits large and very large gathering of more than 100-1000 people) | 2 |
| **Social Gatherings** | Mandates - Medium (e.g., prohibits medium and large gatherings of more than 10-50 people) | 3 |
| **Social Gatherings** | Mandates - High (e.g., prohibits small, medium and large gatherings of people not living in the same household) | 4 |
| **Social Gatherings** | Mandates - Very High (e.g., prohibits all non-essential gatherings outside of household, including gatherings of persons living in the same household) | 5 |
| **Religious Gatherings** | No recommendations or mandates for this domain | 0 |
| **Religious Gatherings** | Recommendations only (e.g., minimize large gatherings of more than 250-500 or recommendations to use social distancing + and use remote services where possible) | 1 |
| **Religious Gatherings** | Mandates - Low (e.g., prohibits large religious of more 100-250 people or occupancy caps of approx. 50% or allows church services if social distancing followed) | 2 |
| **Religious Gatherings** | Mandates - Medium (e.g., prohibiting medium and large religious gatherings with more than 10-50 people or occupancy caps of 25% or below) | 3 |
| **Religious Gatherings** | Mandates - High (e.g., prohibits small, medium and large religious gatherings, except for people living in the same household) | 4 |
| **Religious Gatherings** | Mandates - Very High (e.g., no religious gatherings allowed outside of the household) | 5 |
| **Funerals** | No recommendations or mandates for this domain | 0 |
| **Funerals** | Recommendations only (e.g., minimize large gatherings of more than 250/500 or recommendations to use social distancing + hygiene protocols, and use remote services where possible) | 1 |
| **Funerals** | Mandates - Low (e.g., require social distancing or large occupancy cap) | 2 |
| **Funerals** | Mandates - Medium (e.g., prohibits more than 10-50 attendees at funerals; or occupancy caps of approx. 25% or below) | 3 |
| **Funerals** | Mandates - High (e.g., funeral attendees must live in the same household) | 4 |
| **Funerals** | Mandates - Very High (e.g., prohibiting attendees at funerals) | 5 |
| **Stay at Home/Shelter in Place Orders** | No recommendations or mandates for this domain | 0 |
| **Stay at Home/Shelter in Place Orders** | Recommendations Only (e.g., recommendations to stay at home only for vulnerable populations) | 1 |
| **Stay at Home/Shelter in Place Orders** | Mandates - Low (e.g., only applies to limited groups during specified times, strong social distancing mandate but not a stay at home order) | 2 |
| **Stay at Home/Shelter in Place Orders** | Mandates - Medium (e.g., only applies to higher risk groups) | 3 |
| **Stay at Home/Shelter in Place Orders** | Mandates - High (e.g., broadly applicable shelter in place order with exceptions for essential activities) | 4 |
| **Stay at Home/Shelter in Place Orders** | Mandates - Very High (e.g., all residents stay at home, except for essential activities, plus additional measures to reduce social mobility, a curfew, requiring permits when leaving the home, or limiting only 1 person to leave the home at a time) | 5 |
| **Restaurants** | No recommendations or mandates for this domain | 0 |
| **Restaurants** | Recommendations only (e.g., physical distancing in line, limiting hours, limiting occupancy, or closure) | 1 |
| **Restaurants** | Mandates - Low (e.g., require placing tables 6 feet apart but no occupancy cap) | 2 |
| **Restaurants** | Mandates - Medium (e.g., limiting occupancy, limiting hours of operation, limiting on-premise consumption to outdoors only) | 3 |
| **Restaurants** | Mandates - High (e.g., prohibiting on premises dining, but not prohibiting takeout/delivery) | 4 |
| **Restaurants** | Mandates - Very High (e.g., restaurants ordered closed with no takeaway) | 5 |
| **Bars** | No recommendations or mandates for this domain | 0 |
| **Bars** | Recommendations only (e.g., physical distancing in line, limiting hours, limiting occupancy, or closure) | 1 |
| **Bars** | Mandates - Low (e.g., take steps to require physical distancing in lines or plastic screens in front of registers) | 2 |
| **Bars** | Mandates - Medium (e.g., limiting occupancy, limiting hours of operation, limiting on-premises consumption to outdoors only) | 3 |
| **Bars** | Mandates - High (e.g., prohibiting on premises dining, but not prohibiting takeout/delivery) | 4 |
| **Bars** | Mandates - Very High (e.g., bars ordered closed with no takeaway) | 5 |
| **Indoor Movie Theatres** | No recommendations or mandates for this domain | 0 |
| **Indoor Movie Theatres** | Recommendations only (e.g., physical distancing in line, limiting hours, limiting occupancy, or closure) | 1 |
| **Indoor Movie Theatres** | Mandates - Low (e.g., require physical distancing in lines or plastic screens in front of registers) | 2 |
| **Indoor Movie Theatres** | Mandates - Medium (e.g., moderate limits on occupancy such as 50% capacity or only requiring that groups sit 6 feet apart but no cap on occupancy) | 3 |
| **Indoor Movie Theatres** | Mandates - High (e.g., very limited occupancy (e.g., 25% or less)) | 4 |
| **Indoor Movie Theatres** | Mandates - Very High (e.g., must close to the public and prohibit employees from working on premise) | 5 |
| **Hair Salons/Barber Shops** | No recommendations or mandates for this domain | 0 |
| **Hair Salons/Barber Shops** | Recommendations only (e.g., physical distancing in line, limiting hours, limiting occupancy, or closure) | 1 |
| **Hair Salons/Barber Shops** | Mandates - Low (e.g., require physical distancing in lines or plastic screens in front of registers) | 2 |
| **Hair Salons/Barber Shops** | Mandates - Medium (e.g., limiting occupancy or requiring appointments) | 3 |
| **Hair Salons/Barber Shops** | Mandates - High (e.g., closing some premises to the public, but allowing others to continue to operate with other restrictions in place or allowing employees to continue to work onsite to allow for online-based services) | 4 |
| **Hair Salons/Barber Shops** | Mandates - Very High (e.g., closing all premises to the public and prohibiting employees from working on premise) | 5 |
| **Indoor Gyms** | No recommendations or mandates for this domain | 0 |
| **Indoor Gyms** | Recommendations only (e.g., physical distancing in line, limiting hours, limiting occupancy, or closure) | 1 |
| **Indoor Gyms** | Mandates - Low (ex. require physical distancing in lines or plastic screens in front of registers) | 2 |
| **Indoor Gyms** | Mandates - Medium (e.g., limiting occupancy or hours of operation) | 3 |
| **Indoor Gyms** | Mandates - High (e.g., closing some premises to the public, but allowing others to continue to operate with other restrictions in place or allowing employees to continue to work onsite to allow for online-based services) | 4 |
| **Indoor Gyms** | Mandates - Very High (e.g., closing all premises to the public and prohibiting employees from working on premise) | 5 |
| **Non-Essential Retail Stores** | No recommendations or mandates for this domain | 0 |
| **Non-Essential Retail Stores** | Recommendations only (e.g., physical distancing in line, limiting hours, limiting occupancy, or closure) | 1 |
| **Non-Essential Retail Stores** | Mandates - Low (ex. take steps to require physical distancing in lines or plastic screens in front of registers) | 2 |
| **Non-Essential Retail Stores** | Mandates - Medium (e.g., limiting occupancy or hours of operation) | 3 |
| **Non-Essential Retail Stores** | Mandates - High (e.g., allowing curbside pickup or online order processing; only closing some non-essential businesses) | 4 |
| **Non-Essential Retail Stores** | Mandates - Very High (e.g., closing all premises to the public and prohibiting employees from working on premise) | 5 |
| **Childcare/Early Learning** | No recommendations or mandates for this domain | 0 |
| **Childcare/Early Learning** | Recommendations only (e.g., recommend restrictions on visitors and/or screening at entrance) | 1 |
| **Childcare/Early Learning** | Mandates - Low (e.g., restrictions on visitors, screening at entrance; must close buildings temporarily for cleaning if they have a confirmed case) | 2 |
| **Childcare/Early Learning** | Mandates - Medium (e.g.; reductions on class and/or facility capacity to comply with social distancing) | 3 |
| **Childcare/Early Learning** | Mandates - High (e.g., close except for those serving frontline or essential workers; may require special permit) | 4 |
| **Childcare/Early Learning** | Mandates - Very High (e.g., all childcare/early education facilities forced to close, with only very limited exceptions) | 5 |
| **K-12** | No recommendations or mandates for this domain | 0 |
| **K-12** | Recommendations only (e.g., cancel or reschedule large events) | 1 |
| **K-12** | Mandates - Low (e.g., schools required to cancel large events; schools required to close temporarily for cleaning if they have a confirmed case) | 2 |
| **K-12** | Mandates - Medium (e.g., no in-person instruction at public schools, but other services provided at public schools may continue; either no guidance regarding private schools or recommendation that private schools end in-person instruction) | 3 |
| **K-12** | Mandates - High (e.g., no in-person instruction at public or private K-12 schools, but facilities may still be used for other essential services, such as teachers accessing school facilities to facilitate online learning, childcare, distributing pre-packages lunches) | 4 |
| **K-12** | Mandates - Very High (e.g., no in-person instruction and no admittance to teachers for online learning, but maintenance staff can access for critical facility maintenance) | 5 |
| **Higher Education** | No recommendations or mandates for this domain | 0 |
| **Higher Education** | Recommendations only (e.g., recommendation to cancel or reschedule large events) | 1 |
| **Higher Education** | Mandates - Low (e.g., close buildings temporarily for cleaning if they have a confirmed case; encouraged to examine whether they can continue operating safely; in-person instruction can resume with social distancing) | 2 |
| **Higher Education** | Mandates - Medium (e.g., prohibiting in-person instruction at only public higher education institutions; requiring public higher education institutions to shift to online learning; either no guidance regarding in-person instruction at private higher education institutions or private higher education institutions recommended to end in-person instruction) | 3 |
| **Higher Education** | Mandates - High (e.g., requiring online learning for all public and private higher education institutions, but other services on campus may still continue, such as housing students in dorms, access to teachers to facilitate online learning) | 4 |
| **Higher Education** | Mandates - Very High (e.g., no in-person instruction at private or public higher education institutions; dorms must be vacated; no admittance to teachers for online learning, but maintenance staff can access for critical facility maintenance) | 5 |
| **Nursing Homes** | No recommendations or mandates for this domain | 0 |
| **Nursing Homes** | Recommendations only (e.g., visitor restrictions) | 1 |
| **Nursing Homes** | Mandates - Low (e.g., requiring social distancing in the facility) | 2 |
| **Nursing Homes** | Mandates - Medium (e.g., restriction on number of visitors) | 3 |
| **Nursing Homes** | Mandates - High (e.g., prohibiting non-essential visitors) | 4 |
| **Nursing Homes** | Mandates - Very High (e.g., require full-time "shelter in place" for all facility staff) | 5 |
| **Prisons** | No recommendations or mandates for this domain | 0 |
| **Prisons** | Recommendations only (e.g., physical distancing in facility, visitor restrictions) | 1 |
| **Prisons** | Mandates - Low (e.g., by limiting outside visitors to no more than 3 at a time) | 2 |
| **Prisons** | Mandates - Medium (e.g., only allowing essential visitors, such as attorneys, and banning non-emergency off-site trips); | 3 |
| **Prisons** | Mandates - High (e.g., reduce overcrowding by suspending prisoner transfers; suspending intake of new prisoners from county jails) | 4 |
| **Prisons** | Mandates - Very High (e.g., reduce overcrowding by releasing prisoners who represent low safety risks; prisoners ages 60+ who have release dates within 75 days and whose crimes are nonviolent; commuting sentences for non-violent crimes) | 5 |
| **Voting** | No recommendations or mandates for this domain | 0 |
| **Voting** | Recommendations only (e.g., recommend using existing absentee ballot processes) | 1 |
| **Voting** | Mandate - Low (e.g., extending early voting) | 2 |
| **Voting** | Mandate - Medium (e.g., facilitating mail-in voting by sending out mail-in ballot request forms) | 3 |
| **Voting** | Mandate - High (e.g., requiring that elections be held as mail-in elections, with very limited exceptions) | 4 |
| **Voting** | Mandate - Very High (e.g., postponing elections) | 5 |
